# The supplemental nutrition assistance program and older adults: A scoping review protocol

**DOI:** 10.1371/journal.pone.0321281

**Published:** 2025-04-03

**Authors:** Jessica Soldavini

**Affiliations:** Center for Health Promotion and Disease Prevention and Department of Nutrition, Gillings School of Global Public Health, University of North Carolina at Chapel Hill, Chapel Hill, North Carolina, United States of America; UT Southwestern: The University of Texas Southwestern Medical Center, UNITED STATES OF AMERICA

## Abstract

**Objective:**

The objective of this scoping review is to describe the scope of original published research on SNAP and older adults.

**Introduction:**

Food insecurity among older adults in the United States is a significant public health issue. Participation in the Supplemental Nutrition Assistance Program (SNAP), the largest nutrition assistance program in the United States, by older adults is lower than the overall participation rate.

**Inclusion criteria:**

This scoping review will include original research focused on older adults (aged 60 years and older) and participation in SNAP. Studies should address at least one of the following: examining one or more outcomes related to participation in SNAP, facilitators or barriers to SNAP participation, and/or evaluation of a program, policy or other strategy for influencing SNAP participation.

**Methods:**

Databases to be searched include PubMed, Scopus, CINAHL, and ProQuest. Sciwheel will be used for deduplicating references that will be imported into Covidence for screening and extracting data. Two independent reviewers will screen titles/abstracts followed by full-text articles to assess whether articles meet inclusion criteria and then complete a data extraction template for each included article. A third reviewer will resolve any discrepancies. The findings will be summarized and presented in tables and narrative form.

## Introduction

The population of older adults in the United States is rapidly growing. Data from the United States Census Bureau indicates that between 2010 and 2020, the United States saw the fastest and largest growth in the population aged 65 years or older in any decade since 1880–1890 [1]. In 2020, 16.8% of the population in the United States, or 55.8 million individuals, were aged 65 years or older.

Food insecurity among older adults is a significant public health issue, with over four million households in the United States with adults aged 65 years or older experiencing food insecurity in 2023 [2]. Food insecurity can have a variety of negative consequences for older adults and is associated with malnutrition and poor dietary intake including a lower consumption of vegetables, fruits, dairy, and many micro- and macronutrients compared with older adults who are food secure [3]. Food insecurity is also associated with a higher risk of depression among older adults [4,5]. Individuals who experience food insecurity may need to make the choice between purchasing food or medicine. Studies suggest that cost-related medication nonadherence in older adults may be associated with food insecurity [6].

Nutrition assistance programs can play an important role in addressing food insecurity. The Supplemental Nutrition Assistance Program (SNAP), formerly called Food Stamps, is the largest nutrition assistance program in the United States and provides monthly food benefits to purchase food to low-income households. The program, which is administered by the United States Department of Agriculture (USDA) Food and Nutrition Service (FNS), aims to help low-income households afford healthy food by supplementing their grocery budget. It is important to understand how SNAP participation relates to food insecurity and other outcomes among older adults.

In fiscal year 2022, 38 million individuals were eligible for SNAP, with approximately 34 million (88%) participating [7]. Participation rates varied by demographic group, with individuals aged 60 years or older having lower participation rates with only 55% of eligible individuals participating [7]. Participation rates also varied by living situation, with 71% of eligible individuals aged 60 years or older living alone participating compared with 32% of eligible individuals aged 60 years or older living with others participating [7]. Rates of participation among eligible older adults have been increasing over time, with only 33% of eligible individuals aged 60 years or older participating in fiscal year 2010, however, have consistently been lower than overall participation rates [8]. Participation rates among older adults also vary widely by state. In fiscal year 2018, the states with the highest rates of participation among eligible adults aged 60 years or older were Rhode Island (78%), New York (73%), and Massachusetts (71%) while states with the lowest participation rates among this population were Wyoming (22%), Arkansas (24%), and Utah (28%) [9].

SNAP demonstration projects have been developed to address some of the barriers older adults may face related to participation in SNAP. One example is the Elderly Simplified Application Project, which is a SNAP demonstration project that streamlines the application and certification process for households where all members earn no income and are 60 years or older. In fiscal year 2024, only 22 states plus the District of Columbia participated in this demonstration project [10]. The Standard Medical Deduction Project is a SNAP demonstration project that allows State SNAP agencies to establish a standard medical expense deduction for SNAP households with individuals aged 60 years or older and/or with a disability if out-of-pocket allowable medical expenses for those individuals is over $35 per month. This can help reduce paperwork and administrative burden. In fiscal year 2024, 25 states participated in this demonstration project [10]. The Combined Application Projects (CAPs) are SNAP demonstration projects that streamline the process of applying for SNAP for individuals receiving Supplemental Security Income (SSI), which provides low-income older adults and individuals with disabilities with monthly benefits. The standard model for these projects involves a simplified joint application for SNAP and SSI and the modified model sends simplified SNAP applications using data from the Social Security Administration to eligible households receiving SSI. In fiscal year 2024, seven states participated in standard CAP and 10 participated in modified CAP [10].

Outreach to older adults (individuals aged 60 years or older) around SNAP is a priority area for FNS [7], however, SNAP outreach looks different across states and communities. With a growing population of older adults, low participation rates, and SNAP outreach to this population a priority, it is important to understand facilitators and barriers to SNAP participation as well as potential strategies for increasing participation in this population.

PubMed, PROSPERO, and *JBI Evidence Synthesis* were searched and no completed or in-progress scoping or systematic reviews on SNAP participation and older adults were identified. The type of information available through published research in this area is unclear. Scoping review methodology was selected for this review as the focus is on identifying and summarizing the types of information available through published research on the topic rather than answering a precise research question [11]. This can help inform future research on SNAP and older adults, such as identifying potential outcomes to explore through systematic reviews and outcomes that may benefit from additional studies. The objective of this scoping review is to describe the scope of published original research on SNAP and older adults.

## Research questions

The primary research questions for the scoping review are:

1. What types of outcomes have been examined related to participation in SNAP among older adults?
2. What types of factors have been identified as facilitators or barriers to participation in SNAP among older adults?
3. What types of programs, policies, or other strategies have been evaluated related to influencing participation in SNAP by older adults?

## Inclusion criteria

### Participants

This scoping review focuses on older adults. Older adults are defined as individuals aged 60 years and older to align with how SNAP defines older adults. Studies focused on older adults who define older adults using a different age range can be included if most participants in the sample are 60 years or older. Studies that include a broader age range and include adults aged 60 years or older may be included if they stratify results by age and present findings for this age group. Articles will be excluded if they do not contain individuals aged 60 years or older or include a broader age range and do not report results separately for this group.

### Concept

This scoping review will include studies focused on participation in SNAP. Articles referring to Food Stamps, the original name of the program, will be included. Studies should address at least one of the research questions through examining one or more outcomes related to participation in SNAP, facilitators or barriers to SNAP participation, and/or evaluation of a program, policy or other strategy for influencing SNAP participation.

### Context

There are no limitations related to study context.

### Types of sources

This scoping review will focus on original research. Observational, experimental, and quasi-experimental study designs will be eligible for inclusion. Both quantitative and qualitative studies will be considered. Both peer-reviewed and grey literature, such as reports, working papers, and dissertations may be included if inclusion criteria are met. If a working paper is identified that has a peer-reviewed version published at the time of the review, the articles will be considered duplicates, and the peer-reviewed version of the article will be included. Due to the nature of the research questions and study objective focused on original research, the review will exclude non-original research such as review articles, letters to editors, editorials, and perspectives.

## Methods

The guidelines outlined by the JBI Manual for Evidence Synthesis [11], Preferred Reporting Items for Systematic Reviews and meta-analyses extension for Scoping review (PRISMA-ScR) [12], and Preferred Reporting Items for Systematic Reviews and Meta-analyses Protocol (PRISMA-P) 2015 checklist [13] were used in the development of the study protocol. The PRISMA-ScR (S1 Checklist) and PRISMA-P (S2 Checklist) checklists are included in the supplementary material for this article. This study does not involve human subjects, and therefore, institutional review board approval is not required.

### Search strategy

The databases to be searched for this scoping review include PubMed, Scopus, CINAHL, and ProQuest. An initial limited search of PubMed was conducted to identify relevant articles. The key words, MeSH terms, titles, and abstracts for a sample of these articles were reviewed and used to help develop the full search strategy for PubMed (Table 1). Search strategies from scoping or systematic review articles focused on older adults were also reviewed to further assist with identifying relevant terms related to that population. The PubMed search strategy will be entered into the Polyglot Search Translator from the Systematic Review Accelerator [14] to adapt for Scopus (advanced search), CINAHL, and ProQuest. Reports from organizations known to conduct research and evaluations related to the Supplemental Nutrition Assistance Program, such as Abt Associates, Mathematica, Urban Institute, Food Research & Action Center, Center on Budget and Policy Priorities, Westat, American Public Human Services Association, and Research Triangle Institute, will also be searched to identify additional grey literature on the topic as has been done in prior reviews related to the Supplemental Nutrition Assistance Program [15]. Reference lists of included articles will be searched to identify any potential articles that may have been missed through the initial search. This review will only include articles published in English for feasibility reasons and will not impose any publication date restrictions.

**Table 1:** PubMed Search Strategy.

*Search Terms*
(Elder * [tiab] OR “older adult*”[tiab] OR senior * [tiab] OR aging[tiab] OR aged[tiab] OR aged[Mesh]) AND (“Supplemental Nutrition Assistance Program”[tiab] OR SNAP[tiab] OR “Food Stamp*”[tiab] OR “Electronic Benefit Transfer”[tiab] OR EBT[tiab] OR “food assistance*”[tiab] OR “food assistance*”[Mesh])
*Search Limits*
English Language

### Study/source of evidence selection

Duplicate studies identified through the search will be removed using Sciwheel. The deduplicated reference list will then be imported into Covidence, a collaborative software platform that streamlines the development of scoping, systematic, and other literature reviews, [16] for title/abstract and full-text screening. Each title/abstract will be reviewed by two trained research assistants who will be blinded to the other’s decision. Research assistants will assess whether the titles/abstract appear to meet inclusion criteria and indicate yes (move to full-text screening), maybe (not enough information, move to full-text screening), or no (doesn’t meet inclusion criteria and should be excluded). If any discrepancies arise between reviewers, a third reviewer will make the final decision.

During the full-text screening stage, two trained research assistants will read each article and assess whether the article should be included or excluded based on inclusion criteria. Research assistants will be blinded to the decision made by the other reviewer. A third reviewer will resolve any discrepancies that may arise. During the full-text screening stage, reviewers will report the reason for excluding an article. The results of the literature search and article screening stages will be presented using a Preferred Reporting Items for Systematic Reviews and Meta-analyses extension for Scoping review (PRISMA-SCR) flow diagram [12].

### Data extraction

Two independent reviewers will complete a data extraction template in Covidence for each included article. A third reviewer will resolve any discrepancies. A draft of the items collected through the data extraction template is provided in Table 2. The template will be pilot tested on a small sample of articles and revised as needed during the process of conducting the scoping review. Any modifications made will be described in the scoping review.

**Table 2:** Draft data extraction fields.

Title
Author(s)
Journal
Year of publication
Research question(s) addressed
Study purpose/objectives/aims
Study design
Data sources/data collection
Geographic area
Population, including age range of participants
Sample size
Program, policy, or other strategy evaluated related to influencing participation in SNAP, if applicable
Outcomes examined
Key findings

### Data analysis and presentation

Extracted data will be exported from Covidence as a csv file and used by the study team to summarize findings. Data related to study characteristics and findings of the research questions will be summarized and presented in tables and narrative descriptions. Descriptive statistics will be calculated where relevant.

### Study timeline

It is estimated that it will take approximately 2 months to complete record screening, 1–2 months to complete data extraction, 1–2 months to review results and summarize findings, and 2–3 months to draft the manuscript. At the time of initial submission, the study had not yet started. The goal is to complete the search and import a deduplicated reference list into Covidence by the beginning of February 2025 and start record screening immediately following.

## Discussion

Many older adults in the United States face food insecurity [2], yet SNAP participation rates among older adults are much lower than overall participation rates [7]. This scoping will review will provide an overview of original research published on SNAP participation among older adults, including outcomes associated with participation, facilitators and barriers to participation, and potential strategies for influencing participation rates. A potential limitation of the scoping review is that articles not included in the databases being searched will not be included in the review. This scoping review does not include a critical appraisal of studies for risk of bias, however, this is common practice for scoping reviews [11].

The findings of the study can help inform the development of future systematic reviews as well as identify gaps in research to inform future studies on SNAP participation among older adults. The results of the scoping review will provide an understanding of the types of outcomes that have been assessed related to SNAP participation among older adults. This can help identify which outcomes may have a large enough body of research to conduct a systematic review and which outcomes would benefit from additional research prior to a systematic review taking place. The findings will also help identify whether there may be any potentially relevant outcomes related to SNAP participation that have not yet been examined in older adults. Exploring barriers and facilitators to participation as well as strategies that have been assessed for influencing participation can help with informing future strategies to further explore related to increasing participation rates. Participation rates among older adults in SNAP varies widely from state to state [9] and may be influenced by a variety of factors including programs, policies, and outreach strategies focused on this population. Understanding which programs, policies, and strategies related to SNAP and older adults have been evaluated in different geographic regions may help identify potential strategies to test and evaluate in other areas to assess changes in participation rates. The findings of this scoping review may have programmatic and policy implications in addition to informing future research and evaluation projects.

## Supporting information

S1 Checklist Preferred Reporting Items for Systematic reviews and Meta-Analyses extension for Scoping Reviews (PRISMA-ScR) Checklist. (DOCX)

S2 Checklist Preferred Reporting Items for Systematic review and Meta-Analysis Protocols (PRISMA-P) Checklist. (DOCX)
